# Mosquito-borne alphaviruses in Zambia: Isolation and characterization of Eilat and Sindbis viruses

**DOI:** 10.1016/j.virusres.2025.199604

**Published:** 2025-07-07

**Authors:** Chadwic De’Sean Mears, Koshiro Tabata, Takuma Ariizumi, Bernard M. Hang'ombe, Yongjin Qiu, Hayato Harima, Masahiro Kajihara, William W. Hall, Michihito Sasaki, Hirofumi Sawa, Yasuko Orba

**Affiliations:** a Division of Molecular Pathobiology, International Institute for Zoonosis Control, Hokkaido University, Sapporo, Japan; b Institute for Vaccine Research and Development, Hokkaido University, Japan; c School of Veterinary Medicine, University of Zambia, Lusaka, Zambia; d Africa Centre of Excellence for Infectious Diseases of Humans and Animals, Lusaka, Zambia; e Laboratory of Parasitology, Department of Disease Control, Faculty of Veterinary Medicine, Hokkaido University, Sapporo, Japan; f Laboratory of Veterinary Public Health, Faculty of Agriculture, Tokyo University of Agriculture and Technology, Fuchu, Tokyo, Japan; g Division of International Research Promotion, International Institute for Zoonosis Control, Hokkaido University, Sapporo, Japan; h International Collaboration Unit, International Institute for Zoonosis Control, Hokkaido University, Sapporo, Japan; i National Virus Reference Laboratory, School of Medicine, University College of Dublin, Dublin, Ireland; j Global Virus Network, Baltimore, MD, USA

**Keywords:** Alphavirus, Sindbis virus, Eilat virus, Mosquito, Zambia

## Abstract

Alphaviruses in the family *Togaviridae* include zoonotic arthropod-borne viruses, including Sindbis virus (SINV), chikungunya virus, as well as insect-specific viruses such as Eilat virus (EILV). Previous investigations of alphaviruses in Zambia have identified a novel insect-specific alphavirus, Mwinilunga alphavirus in mosquitoes. Further ongoing surveillance resulted in the isolation of EILV and SINV for the first time in Zambia. Here, these alphaviruses were characterized in terms of growth kinetics in cells, and molecular phylogenetic relatedness to other alphaviruses. Zambian EILV (strain zmq19_M44) exhibited a close phylogenetic relationship with other insect-specific alphaviruses and shared a close nucleotide identity to those of EILV isolate (90.4 %) and Mwinilunga alphavirus (75.5 %). EILV zmq19_M44 attained a saturating titer in C6/36 cells at 6–8-days post infection but was unable to replicate in mammalian cells. Phylogenetic analysis revealed the Zambian SINV (strain zmq17_M115) belongs in Clade D of SINV Genotype 1 along with the Kenyan isolate BONI 584 from Central Africa. The growth of the SINV zmq17_M115 was comparable to that of the prototype SINV strain AR339 in mammalian cells but was statistically different in insect cells. Our findings will contribute to public health measures for the control of alphaviral diseases in Zambia.

## Highlights

• Sindbis virus was isolated for the first time from mosquitoes in Zambia.
• Eilat virus, another insect-specific alphavirus, was also identified in Zambia.
• The potential for zoonotic spillover of Sindbis virus was recognized.

## 1 Introduction

Members in the genus *Alphavirus,* family *Togaviridae* cause a number of diseases, including encephalitis and arthritis, and have different host ranges which include mammals, birds, and insects. While several alphaviruses are associated with human diseases, they remain poorly understood in terms of their mechanism of host specificity, evolution and pathogenesis (Baxter and Heise 2020). Eilat virus (EILV) is an insect-specific alphavirus first isolated from *Anopheles coustani* mosquitoes in Israel (Nasar et al., 2012) and then from *Culex pipiens* in Morocco and *Culex univittatus* mosquitoes in Namibia (Bennouna et al. 2019; Guggemos et al. 2024). EILV displays host restriction characteristics, shown by its ability to only replicate in mosquito cells but not in mammalian or avian cells (Nasar et al. 2012) (Nasar et al. 2015) and is phylogenetically close to viruses of Western equine encephalomyelitis virus (WEE) complex. In Zambia, we previously identified a novel alphavirus, Mwinilunga alphavirus (MWAV) in 2016 (Torii et al., 2018). MWAV is phylogenetically closely related to EILV, and a linear discriminant analysis based on dinucleotide ratios suggest MWAV is an insect-specific alphavirus. Meanwhile, Sindbis virus (SINV) is an Old-World alphavirus which infects birds and mammals and has been used as a model for studying alphavirus replication kinetics (Sokoloski et al. 2012). It is maintained by migratory bird populations and transmitted by ornithophilic mosquitoes to humans which act as dead-end hosts (Adouchief et al. 2016; Lundström, Turell and Niklasson 1992). In the acute phase of SINV infection in humans, the clinical condition includes rash, fever, headache and fatigue. In persistent SINV infection, long term musculoskeletal effects can occur which are accompanied by extensive joint pain. Although migratory birds serve as the main amplifying hosts of SINV, they do not produce any signs of illness despite carrying significant amounts of virus (Hesson et al. 2016). After the first isolation of SINV from *Culex* sp. in Egypt in 1952, it has been subsequently identified in *Culex pipiens, Culex univittatus* (Wills et al. 1985), *Culex torrentium* (Hesson et al. 2015), *Culiseta morsitans* (Bergqvist et al. 2015), *Aedes* sp. (Lundström et al. 2019) *Anopheles* sp. (Jöst et al. 2010) and has disseminated from the African Continent to other areas in the Eastern Hemisphere (Nir et al. 1967; Jalava et al. 2013; Ling et al. 2019). There are six recognized SINV genotypes (Genotype 1–6). Genotypes 2, 3, 5, and 6 are found in Australia, India, New Zealand, and Australia, respectively (Lundström and Pfeffer 2010; Michie et al. 2023). Meanwhile, Genotype 1 has been detected in Central and South Africa, Northern Europe and the Middle East, and Genotype 4 has been reported in Azerbaijan and Western China. Of the 6 genotypes, only Genotype 1 causes human disease (Adouchief et al. 2016; Lundström and Pfeffer 2010). There has been a reintroduction of SINV into Central Africa, but so far this has not been reported in Zambia despite the circulation of other arboviruses and the presence of associated mosquito vectors and intermediate hosts (Orba et al. 2018; Torii et al. 2018; Velu et al. 2021).

In this paper, we have reported the successful isolation of EILV from mosquitoes in Zambia, as well as the isolation of SINV. We have characterized these isolates in terms of their growth properties in mammalian and insect cells and their phylogenetic relatedness to previously reported strains based on whole genomic sequences.

## 2 Materials and methods

### 2.1 Cells and viruses

C6/36 (CRL-1660, ATCC, Manassas, VA) and BHK-21 (RCB1423, RIKEN BRC, Tsukuba, Japan) cells were maintained in Eagle's Minimum Essential Medium (EMEM) containing 10 % fetal bovine serum (FBS) under 5 % CO_2_ at 28 °C and 37 °C, respectively. Vero cells (VERO C1008, CLR-1586) were maintained in Dulbecco's Modified Eagle Medium (DMEM) containing 10 % FBS under 5 % CO_2_ at 37 °C. The SINV AR339 strain (VR-1248) was obtained from the ATCC.

### 2.2 Mosquito collection and alphavirus screening

Mosquito surveillance was conducted in the Western part of Zambia (Mongu district) between 2017 and 2019 with permission from the Excellence in Research Ethics and Science (ERES) converge ethics committee (IRB No: 00,005,948) and University of Zambia Biomedical research ethics committee (REF. NO. 1382–2020) (Orba et al. 2018). In total, 5843 female mosquitoes were captured and divided into 329 pools by species (maximum 40 mosquitoes per pool). The pooled mosquitoes were homogenized in EMEM with 2 % FBS. A part of homogenate was subjected to RNA extraction with Direct-zol RNA Miniprep kit (Zymo Research, Irvine, CA). Pan-alphavirus RT-PCR screens were conducted as described previously (Torii et al., 2018). In brief, RT-PCR was performed with PrimeScript One Step RT-PCR Kit Ver.2 (Takara Bio, Shiga, Japan) using pan-alpha nsP4–6692F (CAYACRYTRTTYGAYATGTCDGC) and pan-alpha nsP4–7152R (GCRTCDATKATYTTBACYTCCAT) with the following condition: 50 °C for 30 min, 94 °C for 2 min, 43 cycles of 94 °C for 30 s, 52 °C for 30 s, 72 °C for 30 s, and 72 °C for 5 min 60 °C. The PCR products (approximately 460 bp) were visualized on agarose gels stained with ethidium bromide and purified on 2 % (w/v) low melting SeaPlaque agarose (Lonza, Sydney, Australia) and the MonoFas DNA Purification Kit I (GL Sciences, Tokyo, Japan). The sequence of the purified PCR products was analyzed by Sanger Sequencing (Big Dye Terminator v3.0 Cycle Sequencing kit) using the ABI Genetic Analyzer 3500 XL (ThermoFisher Scientific, Waltham, MA) and analyzed by Basic Local Alignment Search Tool (BLAST: https://blast.ncbi.nlm.nih.gov/Blast.cgi)

### 2.3 Virus isolation and whole genome sequencing

Either C6/36 or BHK-21 cells were inoculated with the homogenates positive for pan-Alpha virus RT-PCR and cultured for one week. After the second passage of the homogenate-inoculated cells, the supernatants from the cells showing a cytopathic effect (CPE) were subjected to cloning by limiting dilution methods using BHK-21 or C6/36 cells. After 3–6 passages of the cells, the supernatants were subjected to RNA extraction with Direct-zol RNA Miniprep kit. Whole genome sequencing of the purified RNA samples was conducted through the Illumina Miseq platform (Illumina Inc, San Diego, CA) using Nextera XT DNA Library Prep (Illumina, San Diego, CA, USA) followed by sequencing with a MiSeq Reagent Kit v3 (CLC bio, Qiagen, Hilden, Germany). The terminal sequence of viral genome was dteremined by the 5′- and 3′- rapid amplification of cDNA ends (RACE) using SMARTer RACE 5′/3′ kit (Takara Bio) to obtain the terminal sequences as described previously (Torii et al. 2018).

### 2.4 qRT-PCR screening for EILV and SINV

Quantitative RT-PCR (qRT-PCR) screening was performed on all the mosquito pools using the One Step TB Green PrimeScript PCR Kit II (Takara Bio) on a QuantStudio 3 real-time PCR system (ThermoFisher Scientific) with the following protocol (42 °C for 5 min, 95 °C for 10 s, 40 cycles of 95 °C for 3 s 60 °C for 30 s) using the primer sets as follows; zEilat 1067F (CAACCATCTGCGACCAAATGA), zEilat 1237R (TGCTGAATCCTTGTGCTACGG) and zSINV q466F (GCTTCCACAACGATGTTACCT), SINV 603R (GTCGAAGCCAATCCAGTACA). The minimum infection rate (MIR) was calculated by maximum likelihood estimation in the Pooled Infection Rate version 4.0 (Biggerstaff 2005). The confidence interval (CI, 95 %) was indicated in the range between the upper and lower bounds.

### 2.5 Growth kinetics assay

The plaque forming unit (PFU) of SINV M115 initial stock was determined by plaque assay using Vero cells. For plaque assays, Vero cells seeded in 12-well plates were inoculated with serially diluted-culture supernatants for 1 h with intermittent rocking at 37 °C and then overlaid with DMEM containing 2 % FBS and 1 % methyl cellulose in each well. After 48 hrs, the cells were fixed with 10 % formalin and stained with 1 % crystal violet. At the time of infection, the number of cells in each well to be infected was calculated and the multiplicity of infection (MOI) was determined using the formula MOI = PFU /cell number.

The titer of EILV was determined by the median tissue culture infectious dose (TCID_50_) in C6/36 cells. For the TCID_50_ assays, serially diluted-supernatants were mixed with C6/36 cells at a concentration of 1.0 × 10^6^ cells/mL in 96-well plates. Plates were incubated at 28 °C for 8 days, and TCID_50_ titer was determined by the Spearman-Karber method. At the infection experiments, EILV titers were converted to PFU/ml by multiplying the TCID_50_/ml values by 0.7 to calculate the MOI.

For the growth kinetics assays, C6/36 cells in 6-well plates were infected with EILV at a MOI of 0.01 or SINV at an MOI of 0.01 or BHK-21, and similarly, Vero cells in 6-well plates were infected with EILV at an MOI of 0.01 or SINV at an MOI of 0.0001. Culture supernatants were harvested at different time points and subjected to titration by the TCID_50_ assay for EILV or plaque assay for SINV.

The statistical analyses of growth curve difference between two strains were performed by two-way ANOVA with Bonferroni post-hoc test using Prism 9 (GraphPad software, Boston, MA).

### 2.6 Phylogenetic analysis

Genomic sequences of 40 and 54 reference strains of EILV and SINV, respectively, were obtained from GenBank. For phylogenetic analysis of EILV, multiple amino acid sequence alignment of coding regions of non-structural and structural proteins were constructed with EILV_zmq19_M44 (accession number LC869669) and 40 other representative alphaviruses by MUSCLE in MEGA 11 (Tamura, Stecher and Kumar 2021). For phylogenetic analysis of SINV, multiple nucleotide sequence alignment of coding regions was constructed with SINV_zmq17_M115 (accession number LC869668) and 54 reference strains of SINV by ClustalW in MEGA 11 software. Phylogenetic trees were constructed by the Maximum Likelihood method using a GTR+*G* + 1 model with 1000 bootstrap replicates. Phylogenetic trees were constructed by the Maximum Likelihood method using a JTT model with 1000 bootstrap replicates.

## 3 Results

### 3.1 Isolation of alphaviruses from Zambian mosquitoes

To investigate alphaviruses present in mosquitoes in Zambia, we conducted both viral RNA screening and virus isolation. Initial screening using pan-alphavirus RT-PCR, revealed positive results for a pool of *Culex quinquefasciatus* (zmq19_M44, 40 mosquitoes/pool) and a pool of *Anopheles coustani* (zmq19_M27, 30 mosquitoes/pool) captured in Mongu district, Western Zambia in 2019. The sequence of both PCR products found to be identical, showing similarity to the EILV genome nsP4 region. Pan-alphavirus RT-PCR also detected positive signals in a pool of *Culex univittatus* (zmq17_M115, 31 mosquitoes/pool) from the Mongu district in 2017, and the sequence of the PCR product was similar to the genome of SINV (Table 1). EILV or SINV-specific qRT-PCR was subsequently employed on all the mosquito pools collected in Mongu area to check the prevalence of both alphaviruses. It was shown that zmq19_M27 and zmq19_M44 were positive for EILV with a minimum infection rate (MIR) of 1.51 (95 % CI 0.09–7.02) in 538 *Anopheles* mosquitoes and 0.74 (95 % CI: 0.04–3.55) in 871 *Culex* mosquitoes, respectively. The zmq17_M115 pool was positive for SINV with an MIR of 0.42 (95 % CI: 0.02–2.02) in 746 *Culex* mosquitoes (Table 1). Based on the results of the RT-PCR, we selected zmq19_M44 and zmq17_M115 pools for virus isolation. EILV was isolated only in C6/36 cells. Meanwhile, CPE in inoculated cells with lysates from SINV-positive mosquito pools were observed after a second passage in mosquito-derived C6/36 and hamster-derived BHK-21 cells. EILV and SINV in the supernatants were passaged in C6/36 or BHK-21 cells, respectively. Supernatants containing viruses were used for subsequent analysis.

**Table 1:** Screening results for alphaviruses in mosquitoes captured in Mongu, Zambia.

Year	Pool no.	Mosquito no.	Mosquito species	Pan-alpha RT-PCR positive	SINV qRT-PCR positive (Ct)	EILV qRT-PCR positive (Ct)	MIR (95 % CI)*	Virus isolation (Accession no.)
2017 May	49	746	*Culex* spp.	1	1 (22.3)	0	0.42 (0.02–2.02)	SINV zmq17_M115 (LC869668)
	4	11	*Aedes* spp.	0	0	0		
	9	62	*Coquillettidia* spp.	0	0	0		
	8	132	*Mansonia* spp.	0	0	0		
	1	1	*Uranotaenia* spp.	0	0	0		
	2	10	*Aedeomya* spp.	0	0	0		
	65	1444	*Anopheles* spp.	0	0	0		
2018 Aug	19	314	*Culex* spp.	0	0	0		
	1	2	*Anopheles* spp.	0	0	0		
	1	1	*Coquillettidia* spp.	0	0	0		
2018 Dec	39	874	*Culex* spp.	0	0	0		
	3	6	*Aedes* spp.	0	0	0		
	11	102	*Coquillettidia* spp.	0	0	0		
	16	392	*Mansonia* spp.	0	0	0		
	1	3	*Uranotaenia* spp.	0	0	0		
	1	1	*Aedeomya* spp.	0	0	0		
	7	56	*Anopheles* spp.	0	0	0		
2019	37	871	*Culex* spp.	1	0	1 (13.0)	0.74 (0.04–3.55)	EILV zmq19_M44 (LC869669)
	7	21	*Aedes* spp.	0	0	0		
	11	80	*Coquillettidia* spp.	0	0	0		
	9	174	*Mansonia* spp.	0	0	0		
	1	1	*Uranotaenia* spp.	0	0	0		
	1	1	*Aedeomya* spp.	0	0	0		
	26	538	*Anopheles* spp.	1	0	1 (19.5)	1.51 (0.09–7.02)	
Total	329	5843		3	1	2		

⁎ Minimum infection rates (with 95 % Confidence interval) of EILV or SINV.

### 3.2 Whole genome sequence characterization of Zambian SINV and EILV

Using isolated viruses, we determined the whole genome sequences of the Zambian EILV (strain zmq19_M44, EILV M44) with 11,606 nucleotides (nt) and SINV (strain zmq17_M115, SINV M115) with 11,709 nt using next generation sequencing (NGS) and RACE analyses. The genome sequences of these viruses have been submitted to the DDBJ/EMBL/GenBank databases under the accession numbers LC869669 for SINV_zmq17_M115 and LC869669 for EILV_zmq19_M44.

EILV M44 showed 90.4 % nucleotide sequence similarity to EILV EO329 (accession no NC_018615), a strain isolated from *Anopheles coustani* mosquito in Israel (Nasar et al. 2012), and also shares 75.5 % nucleotide identity to MWAV previously identified from mosquitoes in Zambia (Torii et al. 2018) (Table 2). SINV M115 shares 98 % nucleotide sequence identity to SINV BONI_566_KENYA_2013 strain (accession no KY616987) identified from *Aedes ochraceus* mosquito in Kenya (Table 2). The Zambian M115 had >98 % amino acid sequence similarities to the reference sequence of SINV (accession no NC_001547) and Kenyan SINV strains in all viral proteins with exception of nsp3. M115 nsP3 exhibited relatively low sequence identities (<96.4 %) with known SINV strains (Table 2).

**Table 2:** Nucleotide and Amino acid homology of SINV isolate zmq17 M115 and EILV isolate zmq19 M44 to closely related alphaviruses.

	Sequence identity to Zambian Eilat virus isolate zmq19 M44 ( %)									
	Nucleotide	Amino acid								
	Whole genome	nsP1	nsP2	nsP3	nsP4	Capsid	E3	E2	6k	E1
**Strain**	**11,606 nt**	**543 aa**	**809 aa**	**479 aa**	**608 aa**	**256 aa**	**63 aa**	**420 aa**	**55 aa**	**440 aa**
Eilat virus isolate EO329 NC_018615.1	90.44	97.24	96.42	91.45	96.91	95.65	93.65	93.82	96.36	95.22
Mwinilunga alphavirus Zmq16mw21 LC361437.1	75.51	88.21	88.07	69.12	87.95	76.06	80.36	66.02	83.64	77.27

	Sequence identity to Zambian Sindbis virus isolate zmq17 M115 ( %)									
	Nucleotide	Amino acid								
	Whole genome	nsP1	nsP2	nsP3	nsP4	Capsid	E3	E2	6k	E1
**Strain**	**11,709 nt**	**540 aa**	**807 aa**	**549 aa**	**616 aa**	**264 aa**	**63 aa**	**421 aa**	**55 aa**	**438 aa**
Sindbis virus isolate BONI_584_KENYA_2013 KY616985.1	96.49	99.63	98.88	96.37	99.35	98.86	98.44	98.82	100	99.32
Sindbis virus isolate BONI_566_KENYA_2013 KY616987.1	97.96	99.63	98.88	96.37	99.03	98.86	98.84	98.82	100	99.32
Sindbis virus reference isolate NC_001547.1	96.46	98.7	98.2	95.46	99.03	99.62	99.44	98.11	100	99.34

### 3.3 Phylogenetic analysis and genotype assignment

**Fig. 1:**
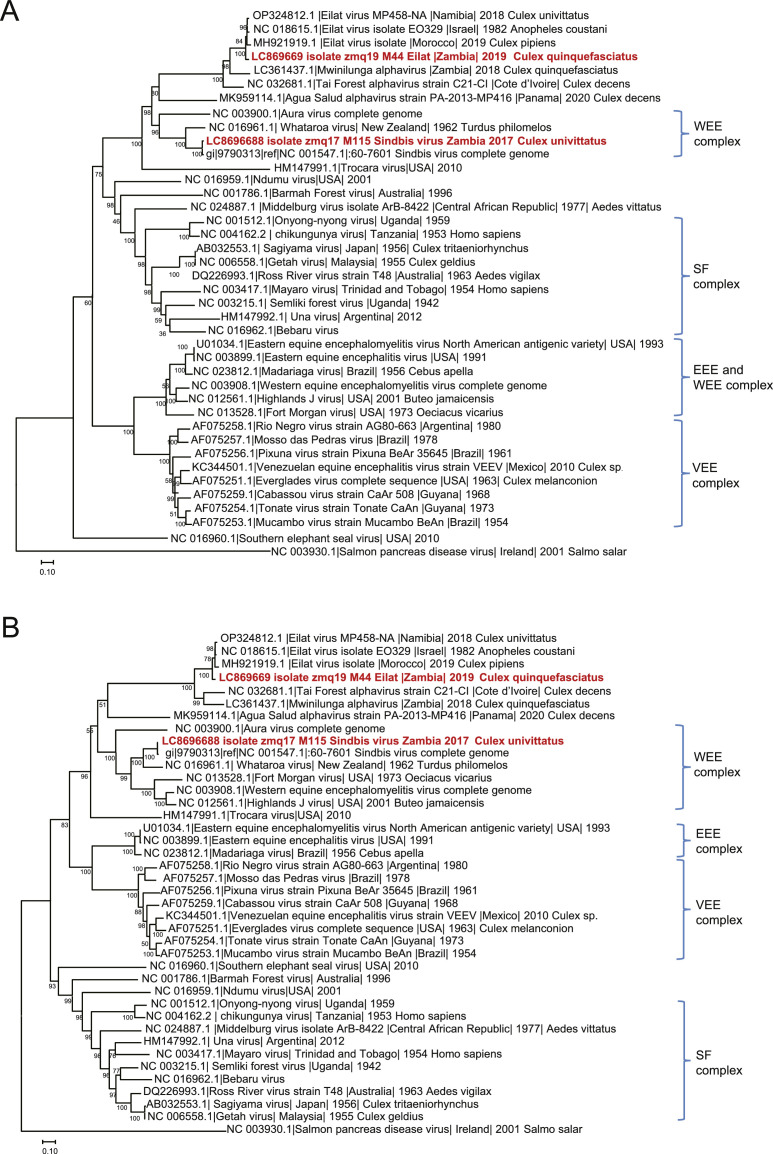
Phylogenetic analysis based on amino acid coding regions of alphaviruses. Phylogenetic analysis of non-structural proteins (A) and structural proteins (B) of alphaviruses. Phylogenetic trees were constructed by the Maximum Likelihood method using a JTT model with 1000 bootstrap replicates. Red text indicates Zambian SINV M115 and EILV M44 isolated in this study. Alphavirus serocomplexes are shown to the right side of the tree. The bootstrap values are shown at the branch node of each clade. The scale bars represent the number of substitutions per site.

Phylogenetic trees were constructed based on coding regions of nonstructural and structural proteins of representative alphaviruses. Zambian EILV M44 formed a cluster with other EILV strains and segregated from other insect-specific alphaviruses such as MWAV and Tai Forest alphavirus in both the non-structural and structural proteins (Fig. 1A and B). The results revealed that two different species of insect-specific alphaviruses are present in mosquitoes living in Zambia.

**Fig. 2:**
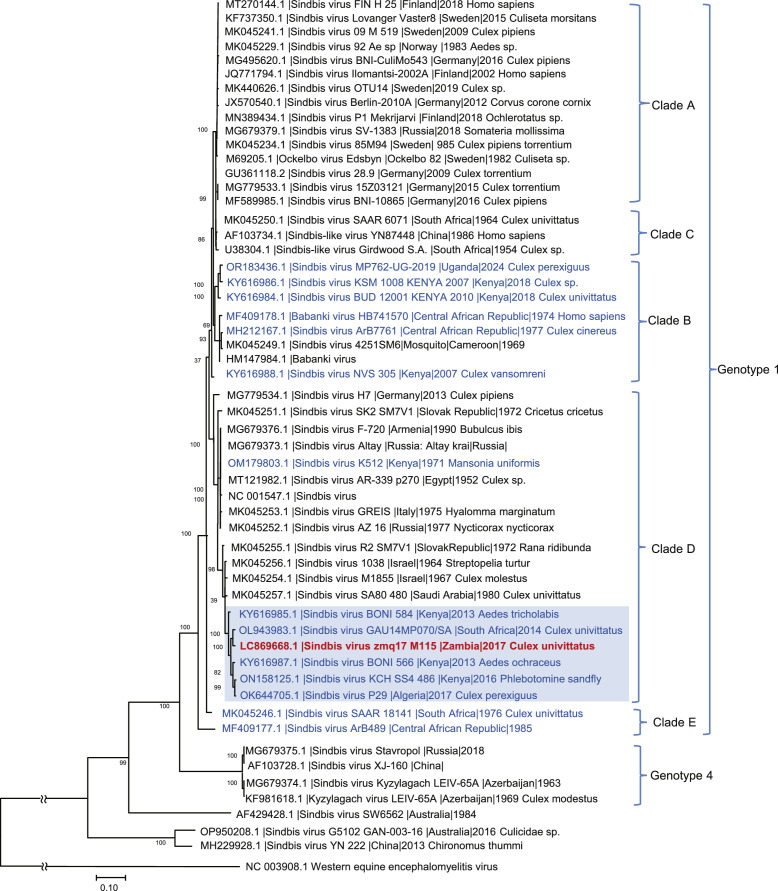
Phylogenetic analysis based on coding sequences of SINV The isolated Zambian SINV M115 in this study is highlighted in red. 54 SINV strains were obtained from GenBank and included in the analysis. The phylogenetic tree was constructed by the Maximum Likelihood method using a GTR+*G* + 1 model with 1000 bootstrap replicates. Genotypes are shown to the right side of the tree. The bootstrap values are shown at the branch node of each clade. The scale bars represent the number of substitutions per site. Red text indicates Zambian SINV M115 and isolated in this study, and blue text indicates African SINV isolates. The cluster including SINV M115 in clade D is highlighted in light blue.

SINV strains consists of 6 genotypes and have an area specific evolutionary history (Lundström and Pfeffer 2010). The phylogenetic analysis of SINV revealed Zambian SINV M115 belongs to the Genotype 1 clade D forming a cluster with SINV isolates from Kenya, South Africa, and Algeria collected from 2013 to 2017 (Fig. 2). Other clusters in the clade D also included the prototype AR339 strain from Egypt and SINV isolates from Middle Eastern countries, as well as Southern and Central Europe, and most of them were found before 1990. The discovery of SINV in Zambia indicated that the distribution of clade D SINVs were expanding widely across Africa in recent years.

### 3.4 Growth kinetics of the isolated Zambian EILV and SINV

**Fig. 3:**
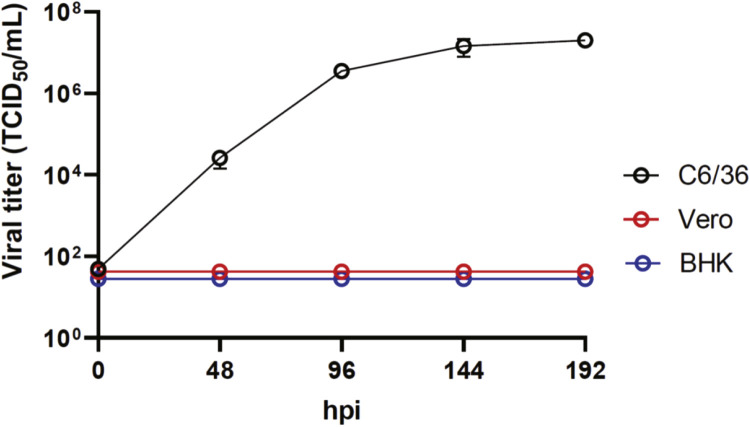
Growth kinetics of Zambian EILV isolate M44 Vero, BHK-21 and C6/36 cells were infected with EILV M44 at an MOI of 0.1. Titers at the indicated time points were determined by TCID_50_. The values shown are mean ± standard deviation (SD) of triplicate studies.

To investigate the in vitro replication properties of the Zambian EILV M44, we examined the growth property of EILV M44 in C6/36, Vero and BHK cells. The growth of EILV M44 was exclusively observed in mosquito-derived C6/36 cells and attained a saturating titer of 10^7^ TCID_50_/mL at 144 to 192 h post infection (hpi), while failing to replicate in mammalian cells, Vero and BHK (Fig. 3). We confirmed that the growth property of EILV M44 is consistent with those of insect-specific alphaviruses (Nasar et al. 2012).

**Fig. 4:**
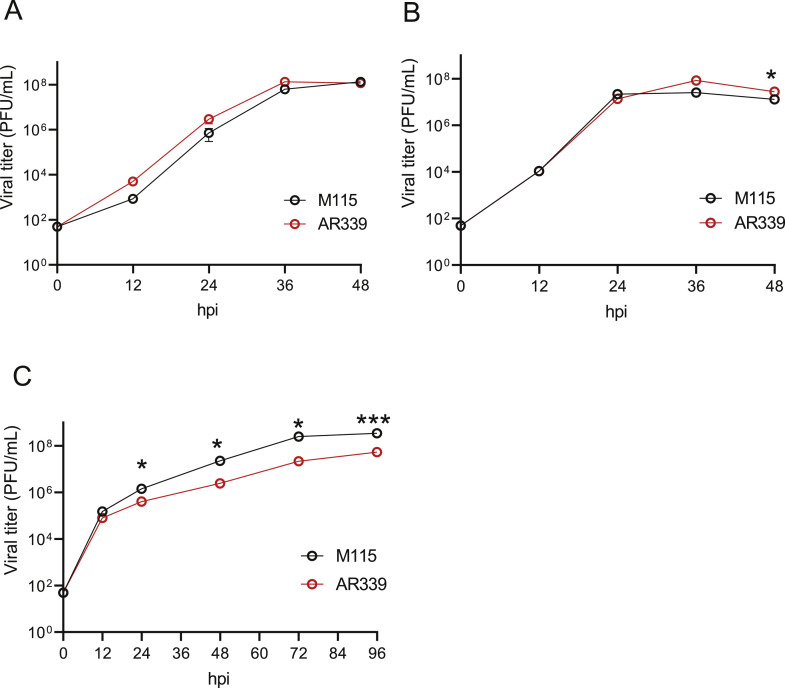
Growth kinetics of Zambian SINV isolate M115 Vero (A) and BHK-21 (B) cells were infected with SINV isolate M115 and AR339 at an MOI of 0.0001. C6/36 cells (C) were infected with SINV at an MOI of 0.01. Titers at the indicated each time point were determined by plaque assay. The values shown are mean ± standard deviation (SD) of triplicate. *p < 0.05, ***p < 0.001 by two-way ANOVA test.

Next, we examined the growth properties of Zambian SINV M115 compared with reference SINV strain AR339. Both SINV M115 and AR339 attained a saturating viral titer of 10^8^ PFU/mL at 36 hpi in BHK-21 and Vero cells (Fig. 4A and B). AR339 reference strain had relatively high growth property in these mammalian cell lines compared to Zambian M115. In the mosquito-derived cell line C6/36, SINV growth kinetics were relatively slower than those in mammalian cells consistent with a previous study (Jose, Taylor and Kuhn 2017). However, the growth of SINV M115 reached 10^8^ PFU/mL at 72 hpi and was statistically higher than that of AR339 (Fig. 4C). These results suggest that the newly isolated SINV M115 strain, obtained from mosquitoes, exhibits greater proliferative properties in mosquito cells that the laboratory-adopted SINV AR339.

## 4 Discussion

In this study, we have reported successful isolation of EILV and SINV from *Culex* mosquitoes from mosquitoes in Zambia for the first time. Our results regarding the in vitro growth characteristics of the two alphaviruses demonstrated properties that correlated well with previously studied properties of these viruses (Sokoloski et al. 2012), (Nasar et al. 2012). Specifically, this reflects the inability of EILV to grow in mammalian cells. Another suspected insect-specific alphavirus, MWAV, has also been found in *Culex* mosquitoes in the neighboring North-Western Province of Zambia (Torii et al. 2018). There is a diversity of known and unknown alphaviruses, and further research, including the possibility of unknown hosts other than mosquitoes for insect-specific alphaviruses, will be of interest in elucidating the ecology and evolution of alphaviruses.

The mechanism of the introduction of EILV into Zambia is puzzling due to the large geographical distance between Zambia, Israel, Morocco,and Namibia, and the limited host range and opportunities for global dissemination of the virus. The EILV M44 strain is approximately 90 % identical at the nucleotide level to other EILV strains, including those from neighbouring Namibia, suggesting that the EILV may circulate in a limited area and maintained independently in mosquitoes in Zambia. However, there remains the possibility that EILV could spread to other regions due to the presence of other unknown hosts or the movement of mosquitoes associated with logistics.

SINV has been demonstrated to exhibit cross-continental spread *via* major bird-migratory routes (Lundström and Pfeffer 2010). The Zambian SINV strain discovered this time is assumed to circulate among birds and mosquitoes across Africa. However, its significance in human cases in African countries remains unclear due to the limited genetic information available on human derived SINV. Our report warrants serious investigation from a global epidemiological perspective on how alphaviruses are spread and furthermore what are the characteristics of insect-specific alphaviruses that enable them to disseminate from their countries of origin and attain global establishment or endemicity. Mongu is located in the Western region of Zambia and shares its border with Angola. In previous reports surveying the risk of arbovirus introduction into Zambia, human travel to other nearby African nations of South Africa, Democratic Republic of Congo and Angola were identified as contributors to the exposure of arboviruses, including Yellow Fever Virus, Zika Virus and Dengue Virus (Velu et al. 2021; Babaniyi et al. 2015). In addition to this, West Nile virus has been detected from mosquitoes captured in the Western Province of Zambia (Orba et al. 2018). Since SINV was also detected from Mongu, the capital of Zambia's Western Province, this suggests that this area may be a focus for arbovirus circulation and warrants further studies regarding surveillance and monitoring as a proactive measure for Zoonosis Control efforts. Furthermore, this effort should take into account wildlife surveillance to record the cross-boundary movement of avian and mammalian intermediate hosts from Zambia to surrounding nations in order to estimate the risk of arbovirus introduction to and from Zambia. It has been suggested that a single introduction of SINV into an immunologically naïve population is enough to lead to the establishment of variants with epidemic potential (Velu et al. 2021; Babaniyi et al. 2015). Sindbis virus is known to cause an illness characterized by fever, rash, fatigue, and muscle pain. It is therefore important to screen patients presenting to primary healthcare facilities with a febrile illness for SINV as a part of the differential diagnosis.

Environmental assessments indicated that the North-western and Western Provinces of Zambia possess ample annual precipitation and humidity to facilitate the proliferation of mosquito species (Masaninga et al. 2014). Thus, the risk of mosquito-borne viruses being introduced and sustained in these regions through enzootic and epizootic cycles is relatively high. Other abiotic factors such as climate change now need to be considered which could directly impact bird migratory routes and mosquito distribution.

## Data

The genome sequences of SINV and EILV have been submitted to the DDBJ/EMBL/GenBank databases and have been assigned accession numbers LC869668 (SINV_zmq17_M115) and LC869669 (EILV_zmq19_M44).

## CRediT authorship contribution statement

**Chadwic De’Sean Mears:** Writing – review & editing, Writing – original draft, Visualization, Validation, Formal analysis, Data curation. **Koshiro Tabata:** Writing – review & editing, Formal analysis, Data curation. **Takuma Ariizumi:** Writing – review & editing, Formal analysis, Data curation. **Bernard M. Hang'ombe:** Writing – review & editing, Resources, Investigation, Data curation, Conceptualization. **Yongjin Qiu:** Writing – review & editing, Investigation. **Hayato Harima:** Writing – review & editing, Investigation. **Masahiro Kajihara:** Writing – review & editing, Investigation. **William W. Hall:** Writing – review & editing, Writing – original draft. **Michihito Sasaki:** Writing – review & editing, Writing – original draft, Visualization, Validation, Data curation. **Hirofumi Sawa:** Writing – review & editing, Writing – original draft, Validation, Supervision, Investigation, Funding acquisition, Data curation, Conceptualization. **Yasuko Orba:** Writing – review & editing, Writing – original draft, Visualization, Validation, Supervision, Investigation, Funding acquisition, Formal analysis, Data curation, Conceptualization.

## Declaration of competing interest

The authors declare that they have no known competing financial interests or personal relationships that could have appeared to influence the work reported in this paper.

## Data Availability

Data will be made available on request.
